# Binderless Faujasite Beads with Hierarchical Porosity for Selective CO_2_ Adsorption for Biogas Upgrading

**DOI:** 10.3390/molecules28052198

**Published:** 2023-02-27

**Authors:** Dina G. Boer, Zahra Asgar Pour, Jort Langerak, Benny Bakker, Paolo P. Pescarmona

**Affiliations:** 1 Chemical Engineering Group, Engineering and Technology Institute Groningen (ENTEG), Faculty of Science and Engineering, University of Groningen, Nijenborgh 4, 9747 AG Groningen, The Netherlands; 2 DMT Environmental Technology, Yndustrywei 3, 8501 SN Joure, The Netherlands

**Keywords:** zeolites, hierarchical porosity, CO_2_ adsorption

## Abstract

Biomethane can be isolated from biogas through selective CO_2_ adsorption. Faujasite-type zeolites are promising adsorbents for CO_2_ separation due to their high CO_2_ adsorption capacity. While commonly inert binder materials are used to shape zeolite powders into the desired macroscopic format for application in an adsorption column, here we report the synthesis of Faujasite beads without the use of a binder and their application as CO_2_-adsorbents. Three types of binderless Faujasite beads (d = 0.4–0.8 mm) were synthesized using an anion-exchange resin hard template. All the prepared beads consisted mostly of small Faujasite crystals, as demonstrated by characterization with XRD and SEM, which are interconnected through a network of meso- and macropores (10–100 nm), yielding a hierarchically porous structure, as shown by N_2_ physisorption and SEM. The zeolitic beads showed high CO_2_ adsorption capacity (up to 4.3 mmol g^−1^ at 1 bar and 3.7 mmol g^−1^ at 0.4 bar) and CO_2_/CH_4_ selectivity (up to 19 at the partial pressures mimicking biogas, i.e., 0.4 bar CO_2_ and 0.6 bar CH_4_). Additionally, the synthesized beads have a stronger interaction with CO_2_ than the commercial zeolite powder (enthalpy of adsorption −45 kJ mol^−1^ compared to −37 kJ mol^−1^). Therefore, they are also suitable for CO_2_ adsorption from gas streams in which the CO_2_ concentration is relatively low, such as flue gas.

## 1. Introduction

Biogas is an environmentally friendly substitute for natural gas [1]. The main components of biogas are CH_4_ (ca. 60 vol%) and CO_2_ (ca. 40 vol%) [2]. The energy density of biogas is directly related to its CH_4_ content. For example, the energy density (lower calorific value) of CH_4_ is 36 MJ m^−3^, while that of biogas with 60 vol% CH_4_ is 20 MJ m^−3^ at STP conditions [3]. Thus, biogas should be upgraded by selective separation of CO_2_ in order to obtain an efficient replacement for natural gas. The upgraded biogas can subsequently be used as a renewable fuel, for example in combined heat and power plants or as vehicle fuel [2]. Using biogas as a replacement for natural gas additionally helps mitigate the emissions of CH_4_, a greenhouse gas with a global warming potential 28 times greater than CO_2_, into the atmosphere from agricultural waste, manure, or landfills [4].

The most commonly used techniques for the separation of CO_2_ from biogas are absorption in a liquid phase, membrane separation, and adsorption on solids. Among these, adsorption using solid sorbents is considered a very attractive separation technology because it is a straightforward process in which no liquid waste is generated [5,6]. Additionally, solid adsorbents typically require less energy for regeneration because CO_2_ is mainly physisorbed on solid adsorbents, whereas it is chemisorbed on liquid absorbents [7]. 

Among the different types of adsorbents, zeolites are of particular interest due to the possibility of tuning their physicochemical properties (e.g., pore size and organization, composition) to optimize their adsorption behaviour. Furthermore, zeolites exhibit high stability and good adsorption capacities (1–7 mmol g^−1^) at low pressure (1 bar) and can be produced at low cost [8]. Faujasites [FAU] are well known as CO_2_-adsorbents because of their high CO_2_ adsorption capacities compared to other zeolites commonly used for this application, such as zeolite A (framework type [LTA], Linde Type A). The high CO_2_ adsorption capacity can be attributed to the low framework density of FAU (and therefore high accessible pore volume) in combination with low Si/Al ratios (particularly for zeolite X) and thus high number of adsorption sites per unit mass of material (Table S1). The FAU framework possesses a supercage that is accessible through a 12-membered ring (12MR) window with apertures of 7.4 Å [9]. Faujasites can be subdivided into two groups: zeolite X, with a Si/Al ratio between 1 and 1.5, and zeolite Y, with Si/Al > 1.5 [10]. Because the Si/Al ratio of zeolite Y is higher, this material has a lower content of Al and, therefore, fewer countercations per unit mass of material. Since the cation sites are the adsorption sites in Faujasites [11], zeolite Y has a lower adsorption capacity compared to zeolite X. 

Typically, zeolites are synthesized as powders. When zeolites are used in an adsorption process, they must be macroscopically shaped to minimize the pressure drop over the adsorption column [12,13]. In order to shape zeolite powders into beads or extrudates, an inert binder material is typically needed. Since the binder is inert, the adsorption capacity per gram decreases by approximately the same percentage as the amount of binder used. Furthermore, to generate a material with sufficient mechanical stability, the zeolite powder, the binder, and possible additives are compressed [14]. This compression may lead to an unfavourable pore size distribution and, therefore, can hinder the diffusion of CO_2_ into the pores. In this work, binderless Faujasite beads with hierarchical porosity were investigated as an attractive alternative. These binderless beads were synthesized using a hard templating method with an anion-exchange resin that was inspired by previous reports of binderless beads based on different zeolitic frameworks (MFI, BEA, LTA) [15,16,17,18,19,20,21,22]. Our method is conceptually different from other approaches that have been reported for the synthesis of FAU binderless beads, which either rely on the granulation of zeolite X in powder form with metakaolin, followed by an alkaline treatment to convert metakaolin into zeolite X [14], or involve the preparation of silica-chitosan hybrid microspheres, followed by hydrothermal treatment in an alkaline solution, after which the chitosan is removed by calcination [23].

This is the first time that Faujasite beads synthesized using a hard templating method have been investigated as CO_2_-adsorbents. The binderless nature of these beads is attractive compared to conventional shaping methods that require a binder, while the hierarchical porosity in which structural meso- and macropores provide accessibility to the zeolitic micropores is expected to be beneficial for CO_2_ adsorption kinetics. The Faujasite beads reached a CO_2_ adsorption capacity of up to 4.3 mmol g^−1^ at 1 bar and a CO_2_/CH_4_ selectivity of up to 19 at partial pressures mimicking biogas, i.e., 0.4 bar CO_2_ and 0.6 bar CH_4_.

## 2. Results and Discussion

A set of binderless Faujasite beads (F1-beads–F3-beads) was synthesized and tested for their applicability in CO_2_ adsorption. For comparison, extrudates were made from commercial zeolite NaY powder and 20 wt% kaolinite. The beads were also compared to commercial NaY in powder form and to Faujasite in powder form (F1-pow), which was obtained as a side-product of the synthesis of the F1-beads. The materials were characterized by XRD, SEM, XRF, and N_2_ physisorption and tested for their CO_2_ and CH_4_ adsorption to evaluate their potential applicability in biogas upgrading. 

### 2.1. Synthesis and Characterization of the Zeolitic Beads

**Figure 1:**
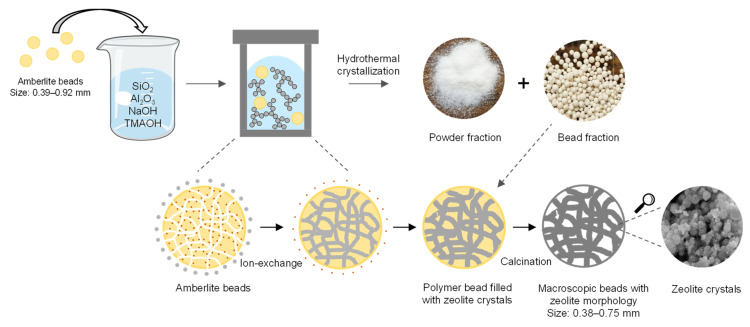
Synthesis of binderless zeolitic beads using an anion-exchange resin as a hard template.

The binderless Faujasite zeolitic beads were synthesized using Amberlite IRA-900, an anion-exchange resin, in a hard templating method. The Amberlite resin has a double role in the synthesis of the beads: it acts as a macroscopic template and thereby shapes the zeolite into a macroscopic bead format, and it provides the beads with a network of meso- and macropores that are generated upon calcination and the consequent removal of the template. The synthesis route that was utilized is shown in Figure 1. It has been suggested that in the initial phase of the synthesis of the beads, the negatively charged zeolite oligomers are formed in the basic reaction mixture and that these are exchanged with the anions of the Amberlite beads [20]. Then, the oligomers undergo condensation and crystallization during the hydrothermal treatment, yielding Amberlite beads filled with interconnected zeolite crystals. The oligomers that do not enter the resin beads but remain in solution are converted into zeolites in powder form as a side-product during the hydrothermal crystallization. Finally, the removal of the resin by calcination endows the material with a hierarchical porous structure in which the meso- and macropores provide access to the micropores of the zeolite crystals. 

The three binderless Faujasite beads were synthesized by adapting a literature method for zeolite Y powder [24] and by varying the ageing and crystallization times in order to optimize the crystallinity and purity of the obtained beads. The average bead size was measured using an optical microscope and was 0.59 mm for the F1-beads and F2-beads and 0.50 mm for the F3-beads (Table 1). 

**Figure 2:**
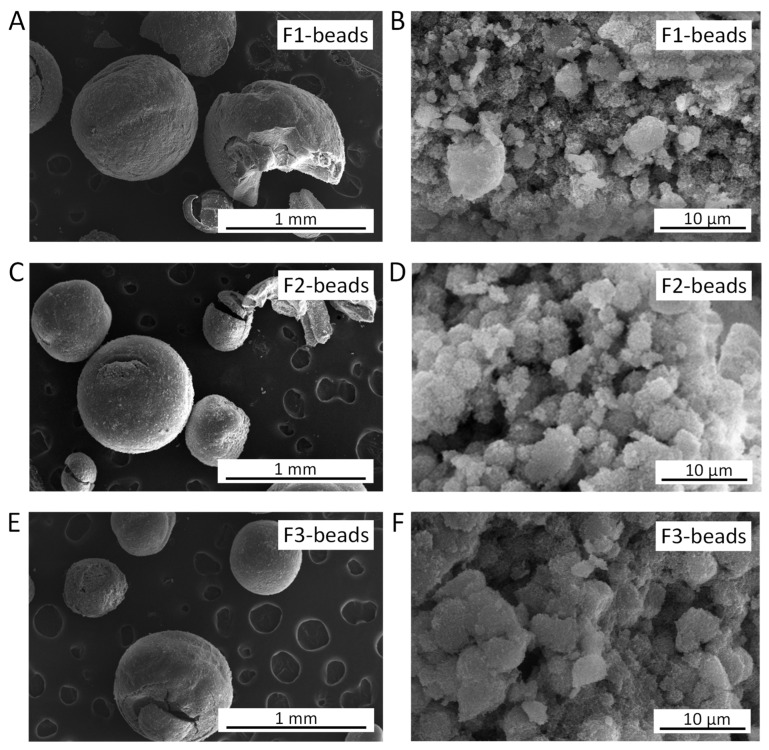
SEM images of: (**A**) F1-beads and (**B**) their interior; (**C**) F2-beads and (**D**) their interior; (**E**) F3-beads and (**F**) their interior.

**Figure 3:**
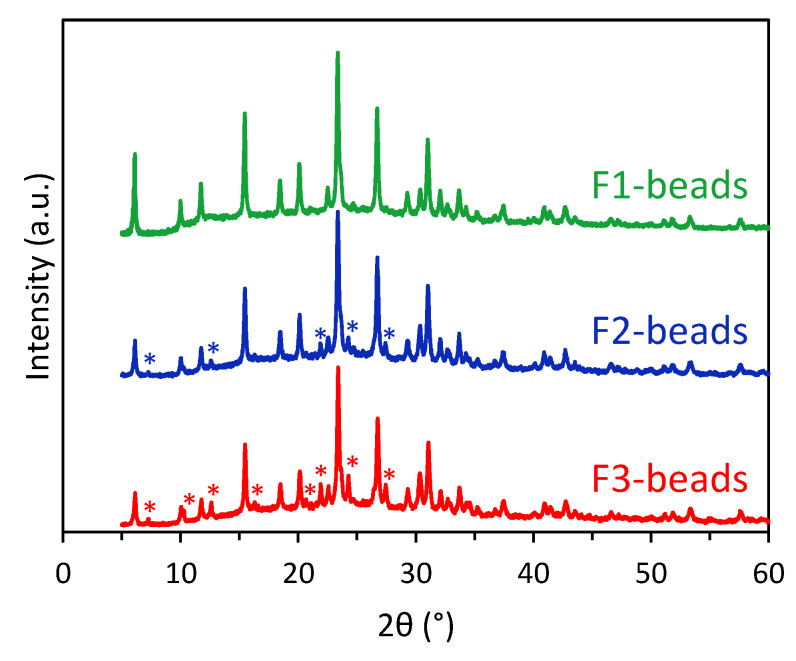
XRD patterns of F1-beads, F2-beads, and F3-beads. Peaks indicated with an asterisk correspond to zeolite with LTA framework (in F2-beads and F3-beads). All the other peaks correspond to zeolite with FAU framework.

SEM images of the F1-beads, F2-beads, and F3-beads revealed that the three materials are similar in appearance: the major fraction consists of intact beads with spherical, though in some cases slightly distorted, shapes. Yet, in all cases, we also observed a few damaged, disfigured, or even completely broken beads (Figure 2A,C,E). Although the beads are similar on a macroscopic level, XRD analysis demonstrated that there are some differences in their crystallinity and purity. All beads primarily consist of the FAU zeolitic framework (Figure 3), and for all beads a broad peak centred at ~23° corresponding to amorphous silica/aluminosilicate was observed (the broad peak is more clearly seen in Figures S1–S3). This is a feature that is commonly observed in zeolitic beads prepared using resin beads as a hard template [15,17,22,25]. Compared to the original synthesis method for zeolite Y powder from the literature [24], in this work the ageing period was prolonged in order to increase the crystallinity of the zeolitic beads. An estimation of the degree of crystallinity of the beads was made by deconvolution of the XRD diffractograms (Table 1, Figures S4 and S5; details about the method used can be found in the SI). The degree of crystallinity of the beads estimated with this approach ranged from 52 to 67%. 

By means of a preliminary, thorough optimization of the length of the two ageing steps (room temperature and 50 °C) and of the hydrothermal crystallization (at 100 °C), we identified some general trends and selected the three methods reported here as the most promising. This optimization study showed that up to 7 days of ageing at room temperature yielded beads that consist exclusively of the FAU framework (as in the case of F1-beads, see Figure 3). Further prolonging this ageing period (8 days at room temperature) did not significantly increase the crystallinity but led to the formation of the competing LTA phase in the beads (as in the case of F2-beads and F3-beads, see Figures S2 and S3). The presence of the LTA phase was minimized by extending the secondary ageing period at 50 °C (compare F2-beads with F3-beads). 

In agreement with the XRD results, the SEM images showed that the interior of the beads generally consists of crystals (Figure 2B,D,F). Although not observed for every bead, generally an amorphous shell covers the beads. In the interior of the beads, a large amount of structural meso- and macropores stemming from the voids between the zeolite crystals were observed (Figure 2B,D,F). This feature was expected because these pores were initially occupied by the template and were thus formed during calcination. Although, in general, the beads consist of an interior of crystals with an amorphous shell, some more peculiar structures have also been observed. In some beads, spherical aggregates of crystals embedded in an amorphous matrix were present (Figure S6). Additionally, a small fraction of empty, typically amorphous shells was observed (Figure S6). 

**Figure 4:**
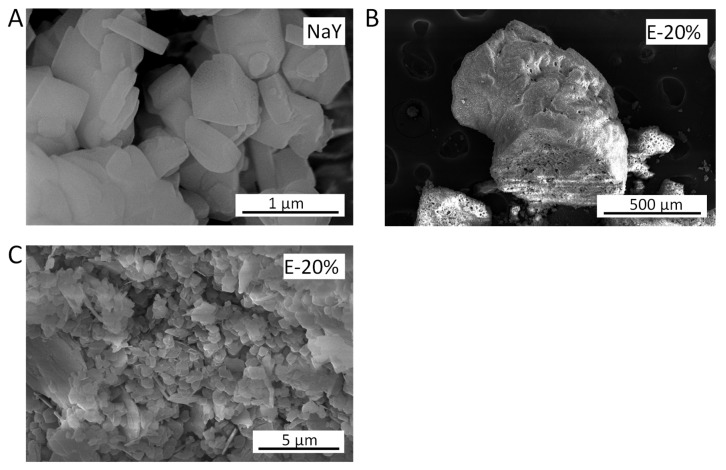
SEM images of: (**A**) commercial NaY powder; (**B**) extrudate from commercial NaY powder (the bottom half was crushed with a spatula to reveal the cross section); (**C**) surface of the extrudate.

In order to have a binder-based material for comparison with the binderless beads, extrudates were made from commercial zeolite Y powder and metakaolin as a binder. The commercial zeolite Y powder used to prepare these extrudates consists of crystals without a defined morphology (Figure 4A). The extrudates displayed an average cross section of 0.68 mm and an average length of 1.4 mm (determined with an optical microscope, see also Figure 4B), and consist of zeolite Y crystals mixed with sheet-like crystals originating from metakaolin (Figure 4C). 

The Si/Al ratios of the beads were determined by XRF spectrometry (Table 2). The F1-beads and F3-beads have a Si/Al ratio similar to that of the commercial zeolite Y powder. The F2-beads possess a lower Si/Al ratio compared to the F1-beads and F3-beads, and, therefore, it is expected that they possess more extra-framework countercations per unit mass compared to the F1-beads and F3-beads. However, the Na/Al ratio of the F2-beads is lower compared to all other materials, leading to an intermediate number of Na-cations in the F2-beads compared to the other beads (Table 2). Although this could suggest that part of the cations are protons (since they cannot be measured by XRF), it is more likely that the Na concentration is underestimated because for all materials, including the commercial NaY powder, an Na/Al ratio < 1 was observed. This may be attributed to the low sensitivity of the employed energy-dispersive XRF for the detection of elements with relatively low atomic masses, such as sodium [26]. It must be noted that the materials, except for the commercial zeolite Y powder, are mixtures of either Faujasite and the amorphous phase (F1-beads-F3-beads and F1-pow) or zeolite Y and kaolinite (Extrudates E-20%). Since the measured Si/Al ratio is that of the whole sample, this value can differ from the Si/Al ratio of the zeolite framework alone. Previous work also showed that the Si/Al ratios of similar zeolite Y beads calculated from XRF data are in good agreement with those calculated from ^29^Si MAS NMR [22]. 

**Figure 5:**
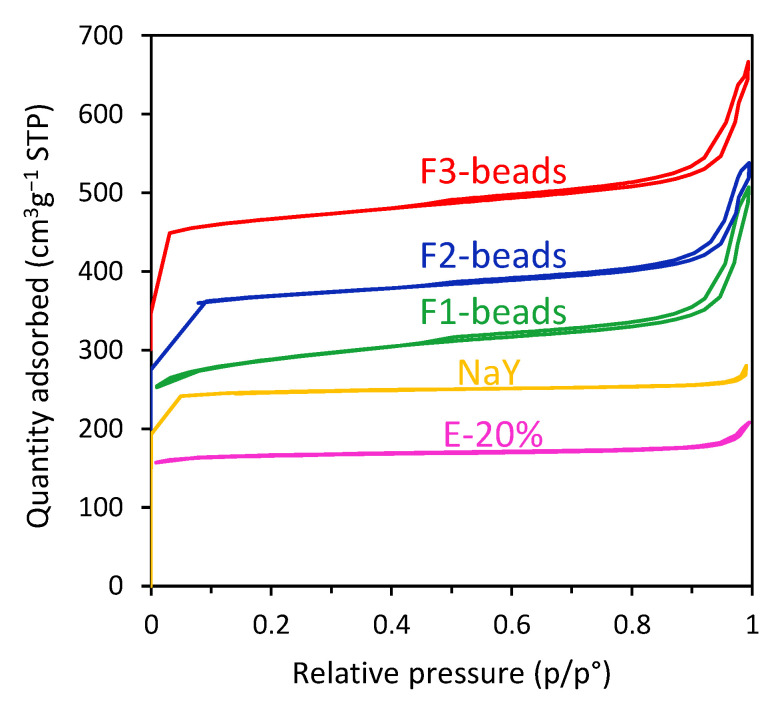
N_2_ physisorption isotherms for F1-beads–F3-beads, commercial zeolite Y powder (NaY), and extrudates of commercial zeolite Y (E-20%). The F1-beads isotherm is offset by 100 cm^3^ g^−1^, the F2-beads isotherm is offset by 200 cm^3^ g^−1^, and the F3-beads isotherm is offset by 300 cm^3^ g^−1^ along the vertical axis to facilitate their visualization.

The surface properties of the beads, F1-pow, the commercial zeolite Y powder and the extrudates were determined using N_2_ physisorption (Figure 5), which allows characterizing the mesopores and the relatively large micropores of this material. For all materials, a sharp increase at p/p^0^ < 0.05 was observed. This indicates the presence of micropores. Additionally, for all the beads, a hysteresis loop was observed from p/p^0^ ≈ 0.45 to p/p^0^ = 1, which indicates the presence of meso- and/or macropores. The micropore volume of the commercial zeolite powder was higher than that of the synthesized beads. This could be explained by its superior crystallinity (Figure S7). The pore size distribution and the micropore volume were similar for all the beads (Figure S8, Table 2), which all possessed high BET surface area (> 500 m^2^ g^−1^) and total pore volume (≥ 50 cm^3^ g^−1^). The F1-beads possessed higher BET surface area and mesopore volume compared to the other beads. This difference is attributed to the presence of FAU as the only zeolitic framework in F1-beads, whereas for F2-beads and F3-beads trace amounts of the LTA framework were also observed. LTA zeolites are small-pore zeolites, and if prepared with a low Si/Al ratio the presence of a large number of countercations per unit mass limits the available pore volume and surface area [25,27]. Whilst the extrudates (E-20%) possess almost no meso- and/or macropores, all the synthesized beads show a large amount of meso- and macropores, with the pore size being estimated to be in the 10–100 nm range (Table 2, Figure S8). This is in agreement with previous reports of beads with different compositions but prepared using the same Amberlite resin as a hard template [22,25,28]. This secondary pore structure in our beads (also shown by SEM, see above) is a desirable property for CO_2_ adsorption processes, as it leads to a hierarchical porous structure in which the micropores are accessible through a network of meso- and macropores. This is expected to facilitate the diffusion of CO_2_ into the beads and thus lead to fast adsorption/desorption kinetics. 

F1-pow is the powder side-product of F1-beads. For this powder, a very high BJH pore volume is observed, as well as a hysteresis loop at high relative pressure in the N_2_ physisorption isotherm (Table 2, Figures S9 and S10). While this feature corresponds to pores in the mesopore range, it actually stems from intercrystalline voids, e.g., the non-structural porosity originated from the volume between the individual zeolite crystals. This is thus inherently different from the mesoporosity in the zeolitic beads, which also originates from the spaces between the zeolite crystals but is structural, as the crystals in the beads are interconnected to each other and thus generate the scaffold holding together the macroscopic spherical structure.

The yields of the beads and of the zeolites in powder form obtained as side-products are given in Table 1. The relative yield of beads was only 6–7.5% of the total yield (beads + powder). This low yield likely originates from the diffusion limitations of the zeolitic oligomers into the Amberlite beads. Future work should be focused on retarding the growth of the oligomers, thereby enabling their diffusion into the Amberlite beads and eventually leading to a higher yield of zeolitic beads. Even though the relative yield of the beads was low, the powder side-product consists of pure Faujasite nanocrystals (about 30–70 nm), which is a valuable material that can be used in many catalytic applications [29,30,31] (Figures S11–S15).

Another aspect of the beads that should be improved in future work is their mechanical stability. The mechanical stability of the beads was lower compared to binderless LTA beads synthesized with a similar method and to commercial binder-containing zeolite LTA beads [25], based on pressing the beads with a spatula. We attempted to quantify the mechanical stability of the F1-beads–F3-beads by means of compression tests [25], but the results were inconclusive. This is probably due to the lower mechanical stability and the smaller bead size of the F1-beads–F3-beads compared to the previously reported LTA beads, leading to less marked signals in the displacement-load graphs. Future optimization of the synthesis method by enhancing the diffusion of the zeolite oligomers into the Amberlite beads (vide supra) could also increase the mechanical strength of the binderless beads since the Amberlite template is probably not completely filled, as evidenced by the cracks in the beads (Figure 2). 

### 2.2. Application of the Zeolitic Beads for CO_2_ Adsorption

The prepared Faujasite beads were tested as CO_2_-adsorbents by comparing their CO_2_ adsorption capacity to their CH_4_ adsorption capacity, with the purpose of evaluating their potential in the separation of CO_2_ from CH_4_ in the context of biogas upgrading. The adsorption mechanism of CO_2_ on Faujasite zeolites has been reported previously [11,32,33,34,35,36]. In Faujasites in the Na-form, CO_2_ mostly interacts with a single Na^+^ site [11,32,33,34,35,36]. In the case of zeolite Na-X, CO_2_ interacts preferably with cations in site III’ (in the supercage, close to one of the 4MRs of the supercage), though at higher loading it also interacts with cations in site II (in the supercage, in front of the 6MR of the sodalite cage). For zeolite Na-Y, site III’ is not occupied by cations, and, therefore, CO_2_ interacts only with cations in site II [11,33,34,35,36].

**Figure 6:**
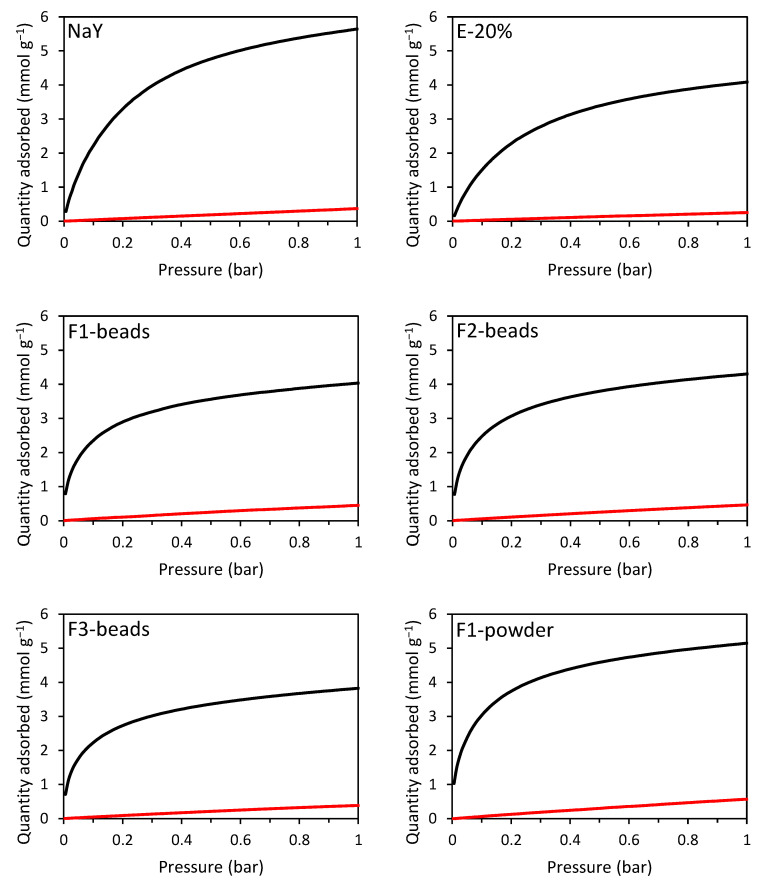
CO_2_ (black) and CH_4_ (red) adsorption isotherms of F1-beads–F3-beads, F1-pow, commercial zeolite Y powder (NaY), and extrudates from commercial zeolite Y with 20 wt% binder (E-20%).

The CO_2_ and CH_4_ adsorption capacities were measured at room temperature in the 0–1 bar range for the F1-beads-F3-beads, F1 powder side-product (F1-pow), commercial zeolite Y powder (NaY), and extrudates from commercial zeolite Y (E-20%, Figure 6 and Table 3). The commercial zeolite Y powder displayed the highest CO_2_ adsorption capacity (5.6 mmol g^−1^), which is similar [37] or slightly higher [38,39] compared to values reported in the literature for zeolite Y powder. However, zeolites in powder format are not suitable for application in commercial pressure swing adsorption equipment since this would lead to a large pressure drop over the adsorption column [12,13]. Our beads inherently overcome this limitation without requiring the use of a binder that, on the other hand, is necessary to prepare conventional extrudates from zeolite in powder form. 

The extrudates prepared in this work consist of 80 wt% commercial zeolite Y powder and 20 wt% binder. Because the binder is inert, the CO_2_ adsorption capacity of the extrudates was expected to be at least 20% lower than that of the powder. The decrease in adsorption capacity compared to the zeolite powder was actually larger (28%), which suggests that the binder also caused partial blocking of the Faujasite micropores [13]. The same trend was observed for the CH_4_ adsorption capacity (Figure 6 and entries 1 and 2 in Table 3). 

All the beads showed comparable CO_2_ adsorption capacity to the extrudates, and in the case of F2-beads, a slightly higher CO_2_ adsorption capacity was observed (4.3 mmol g^−1^ at 1 bar). The results in terms of CO_2_ adsorption capacity can be rationalized on the basis of the physiochemical features of the materials. Well-defined Na^+^ sites in the crystalline microporous framework of the zeolite are the expected sites on which the adsorption of CO_2_ takes place [11]. Therefore, a combination of high Na-content, a high degree of crystallinity, and a large specific surface area and micropore volume is desired for application as a CO_2_-adsorbent. Though the Si/Al ratio of the F2-beads was lower than that of the other beads, the amount of Na per unit mass measured by XRF was lower than that of the F1-beads due to its relatively low Na/Al ratio (Table 2). Yet, this material displayed the highest degree of crystallinity and the largest micropore volume among the beads (Table 1 and Table 2), thus accounting for the higher CO_2_ adsorption capacity displayed by the F2-beads compared to the F1-beads (4.0 mmol g^−1^ at 1 bar). This is in agreement with previous work, in which a higher degree of crystallinity was correlated to a higher CO_2_ adsorption capacity [25]_._ The F3-beads possess the lowest micropore volume, the lowest degree of crystallinity, and the lowest Na-content among the studied beads, which explains their lower CO_2_ adsorption capacity (3.8 mmol g^−1^ at 1 bar) compared to the F1-beads and F2-beads. Though the interpretations provided above are likely to be correct at the qualitative level, caution is advised in the discussion of the observed CO_2_ adsorption capacities on the basis of the Na-content and the related Si/Al and Na/Al ratios measured by XRF because these elemental analyses refer to the whole material and, therefore, do not necessarily reflect the Na-content, Si/Al, and Na/Al ratios in the zeolitic part of the materials (due to the presence of an additional amorphous phase in the beads, vide supra). 

The commercial zeolite Y powder displayed significantly higher CO_2_ adsorption capacity compared to all the beads, despite having a lower content of Na compared to F1-beads. This is ascribed to the larger specific surface area and micropore volume displayed by the commercial NaY zeolite and to its superior crystallinity compared to that of the zeolitic beads (compare Figure 3 to Figure S7). This is in line with previous work, in which the presence of an amorphous silica/aluminosilicate phase in zeolitic beads has been correlated to a decrease in CO_2_ adsorption capacity [25]. 

The fact that the CO_2_ adsorption capacity is determined by a combination of factors is further illustrated by the CO_2_ adsorption capacity of F1-pow. Although the crystallinity (75%), the specific surface area, and the micropore volume of F1-pow were considerably lower than those of the commercial zeolite powder, the CO_2_ adsorption capacity (5.2 mmol g^−1^ at 1 bar) was only slightly lower. This is ascribed to the lower Si/Al ratio of F1-pow (1.5) compared to the commercial powder (2.7) and the related higher Na-content (Table 2), which partially counterbalances the effect of the other parameters. Though F1-pow displayed a lower adsorption capacity compared to the NaY powder, it still outperformed all the Faujasite beads. This is attributed to its higher degree of crystallinity, larger micropore volume, and higher Na-content compared to the beads. However, as F1-pow is a powder, it is not suitable for direct application in an adsorption column since this would lead to a large pressure drop over the column. On the other hand, the Faujasite beads are macroscopically shaped and, therefore, can be utilized directly in an adsorption column.

Notably, the CH_4_ adsorption capacity of all the beads and F1-pow was relatively high compared to that of the commercial zeolite Y powder and the extrudates (Table 3, Figure 6), leading to a lower CO_2_/CH_4_ selectivity for the beads and F1-pow. We hypothesize that this is due to the relatively large individual crystals in the commercial zeolite powder and, therefore, also in the extrudates compared to those in the beads (Figure S16) and the powder side-product (Figure S15). This may cause diffusion limitations within the crystals, which are expected to be more pronounced for CH_4_ than for CO_2_ due to the larger kinetic diameter of the former (3.8 Å and 3.3 Å, respectively). Among the zeolitic beads, the F3-beads gave a slightly higher CO_2_/CH_4_ selectivity (CO_2_/CH_4_ = 19).

Another notable difference between our materials and those based on commercial NaY zeolite is that all the beads and F1-pow displayed a steeper increase in the CO_2_ adsorption isotherm in the low-pressure domain (<0.1 bar) compared to the commercial zeolite powder. A steeper increase in the adsorption isotherm indicates a higher enthalpy of adsorption, as demonstrated by calculating the enthalpy of adsorption for F2-beads (ΔH_ads_ = −45 kJ mol^−1^) and NaY (ΔH_ads_ = −37 kJ mol^−1^) (Figures S17–S20). The enthalpy of adsorption is an indication of the strength of the interaction between CO_2_ and the adsorbent. Though we do not yet have sufficient elements to fully understand the origin of the higher enthalpy of adsorption for our materials, we put forward two hypotheses that could explain the observed difference in the strength of the interaction between CO_2_ and the adsorbent: 

(i) In the F2-beads and the F3-beads, a small amount of LTA zeolite is present, which is known to display a higher enthalpy of adsorption (around 48 kJ mol^−1^) [40] compared to FAU zeolite.

(ii) F1-pow displayed a Si/Al ratio of 1.5 and should thus be considered a type X Faujasite instead of a type Y Faujasite. Additionally, the actual Si/Al ratio of the zeolite phase in F1-beads-F3-beads and F1-pow may be lower due to the presence of an amorphous phase [25]. If the actual Si/Al ratio is ≤1.5, the Faujasite is considered a zeolite X instead of Y. In zeolite Y, cations are only present in site II, while in zeolite X cations are also present in site III’. Interaction of CO_2_ with cations in site III’ leads to a higher enthalpy of adsorption for zeolite X compared to zeolite Y [34].

A high enthalpy of adsorption is a particularly useful feature for applications in which the partial pressure of CO_2_ is low [41]. For example, in flue gas streams the partial pressure is about 0.15 bar, and at this partial pressure our beads showed a similar CO_2_ adsorption capacity to the commercial powder. F1-pow even outperformed the commercial powder at this partial pressure with a CO_2_ adsorption capacity of 0.6 mmol g^−1^. 

## 3. Materials and Methods

### 3.1. Materials

Amberlite IRA-900 in chloride form (particle size 0.39–0.92 mm), natural kaolinite (Al_2_O_3_∙2SiO_2_∙2H_2_O), silica gel (SiO_2_, high purity grade, 230–400 mesh particle size), sodium aluminate (NaAlO_2_), and tetramethylammonium hydroxide solution (TMAOH, 25 wt% in H_2_O) were purchased from Sigma-Aldrich. Sodium hydroxide (NaOH, 98%) was purchased from Boom. Zeolite Y (CBV 100) was purchased from Zeolyst. The H_2_O used in this work was always MilliQ grade.

### 3.2. Synthesis of the Zeolitic Beads

The method for the synthesis of the zeolitic beads is based on a synthesis protocol for zeolite Y powder from the literature [24], which was adapted to include the use of the resin beads as a hard template [22]. 2.0 g of NaOH was added to 15.0 g of deionized H_2_O and 12.0 g of TMAOH (25 wt%) in the Teflon liner of a 100 mL stainless steel autoclave. After the dissolution of NaOH, 1.54 g of NaAlO_2_ was added to the reaction mixture. The mixture was stirred using a magnetic stirring bar at 500 rpm at room temperature until complete dissolution of the solids (2 h). The stirring speed was reduced to 200 rpm, 3.0 g SiO_2_ (silica gel) was gradually added, and the resulting mixture was stirred for 30 min. Then, 1.67 g of Amberlite IRA-900 was added to the silicoaluminate mixture. The molar composition of the reaction mixture was 1 NaAlO_2_: 2.7 SiO_2_: 2.7 NaOH: 1.8 TMAOH: 71 H_2_O. After mixing for 1 min, the autoclave was closed, and the reaction mixture was aged statically at room temperature for 7 or 8 days (see Table 4). The autoclave was then placed into an oven at 50 °C for a secondary ageing period of 3–5 days (see Table 4). Afterwards, the temperature was increased to 100 °C for the static hydrothermal crystallization for 2–4 days (see Table 4). After cooling down to room temperature, the reaction mixture was filtered over a Büchner funnel and washed with deionized H_2_O until the pH was 8. The solids were dried overnight at room temperature. This procedure yielded a mixture of zeolite powder and polymer beads filled with zeolite. The beads were separated from the powder by sieving. Subsequently, the beads and the powders were calcined using the following programme: heating at 3 °C min^−1^ to 200 °C, 6 h at 200 °C, heating at 2 °C min^−1^ to 600 °C, 6 h at 600 °C. The obtained beads were labelled as F1-beads–F3-beads corresponding to the synthesis parameters in Table 4, and the corresponding powder side-products were labelled F1-pow–F3-pow. 

From preliminary studies (data not shown), a ratio of about 1: 20 between Amberlite beads and the synthesis mixture appeared to be optimal. At this ratio, most of the beads were filled with zeolite (as evidenced by the colour change from orange/brownish for the empty beads to white/yellowish for the filled beads), and good crystallinity could be achieved. Increasing the ratio between Amberlite beads and the reaction mixture was investigated with the purpose of increasing the yield of beads (relative to the powder product), but typically resulted in unfilled beads and/or a decrease in crystallinity.

The framework density and the accessible volume and area of FAU, as calculated based on literature data [9,42], are provided in Table S1.

### 3.3. Extrudates from Commercial Zeolite Y Powder

Extrudates were prepared using a Caleva Multi Lab apparatus. 5.0 g of commercial zeolite Y and 1.25 g of kaolinite were mixed for 5 min. 3.6 mL of H_2_O was added dropwise during the first 2 min of the mixing time. The powder was then transferred into the extruder, and spaghetti-like extrudates were formed. The circular openings of the extruder die contained a diameter of 0.75 mm. The extrudates were dried overnight at 100 °C and then calcined at 600 °C for 6h with a heating rate of 3 °C min^−1^. The spaghetti-like extrudates had an average diameter of 0.68 ± 0.03 mm and were cut into small cylinders with an average length of 1.40 ± 0.37 mm. The size range of the extrudates was chosen to be similar to that of the binderless zeolitic beads in order to minimize differences in CO_2_ adsorption capacity caused by the diffusion length within the macroscopic adsorbents.

### 3.4. Characterization

Powder X-ray diffraction (PXRD) patterns of all samples were measured in the range 5–60° with a step size of 0.02° on a Bruker D8 Advance diffractometer with Cu Kα1 radiation (λ = 1.5418 Å) under 40 kV and 40 mA, with a slit-width of 2 mm. The beads were ground into a powder before the PXRD measurements. Energy-dispersive X-ray fluorescence (XRF) measurements were performed on an Epsilon 3^XLE^ spectrometer from PANalytical to determine the elemental composition of the samples. The samples (powders or beads) were measured in a plastic cup with 6 μm mylar film. All samples were measured in duplicate; the values reported are the average of both measurements and their standard deviation. The fundamental parameter method was used for quantification. The elements were assumed to be in their oxide forms and the sum of the resulting concentrations was normalized to 100 %. Nitrogen physisorption measurements were conducted on a Micromeritics ASAP 2420 apparatus at −196 °C. The Brunauer–Emmet–Teller (BET) method was employed to calculate the specific surface area. The pore size distribution and the mesopore volume were calculated using the Barrett–Joyner–Halenda (BJH) model (from the desorption branch). The micropore volume was calculated using the T-plot method. The average bead and extrudate size were calculated using a VHX-7000 Keyence digital microscope with a sample size of 40 beads or extrudates. The bead or extrudate size was reported as average size (mm) ± standard deviation (mm). The surface morphology of the beads and extrudates was investigated by scanning electron microscopy (SEM) on an FEI NovaNano SEM 650 machine. CO_2_ and CH_4_ adsorption isotherms were obtained at 24 °C using a Micromeritics ASAP 2020 apparatus. Prior to the analysis, the samples were degassed for 10 h under a vacuum at 350 °C to remove H_2_O and other possible adsorbed species. The method used for calculating the enthalpy of adsorption [43] is provided in the SI. 

## 4. Conclusions

Hierarchically porous beads containing Faujasite [FAU] zeolite crystals were synthesized for application as adsorbents of CO_2_ from biogas. The beads were structured and shaped using a commercial and inexpensive ion-exchange resin as a hard template. Three types of beads were synthesized, all of which consisted mainly of the FAU framework but also contained an amorphous silica/aluminosilicate phase. Additionally, for some of the beads, a trace amount of LTA zeolite was present. The obtained zeolitic beads had a diameter in the 0.4–0.8 mm range and were characterized by a hierarchical network of meso- and macropores that allowed access to the zeolitic micropores. These structurally porous features were well reproducible when repeating the synthesis.

The binderless beads were compared with extrudates made from commercial zeolite Y. The CO_2_ adsorption capacities of the different beads (3.8–4.3 mmol g^−1^) at 1 bar were comparable to, and for one material (F2-beads) higher than that of the extrudates (4.1 mmol g^−1^). Notably, our beads exhibited a steeper adsorption isotherm at low relative pressure compared to the commercial zeolite powder and thus a higher enthalpy of adsorption (ΔH_ads_ = −45 kJ mol^−1^ for our F2-beads compared to ΔH_ads_ = −37 kJ mol^−1^ for the commercial powder). This indicates that the interaction of CO_2_ with our beads is stronger than with the commercial zeolite. This is a particularly useful characteristic for CO_2_ adsorption from gas streams with relatively low CO_2_ concentrations, such as flue gas.

The CO_2_/CH_4_ selectivity of the extrudates was superior to that of the beads (29 and 19, respectively). Since the extrudates were made from commercial zeolite Y powder, which had relatively large individual crystals (0.35–0.75 mm), it was hypothesized that the diffusion limitations of CH_4_ within these crystals caused the increased selectivity observed with the extrudates. The beads consist of much smaller zeolitic crystals (30–70 nm) and, therefore, experienced less marked diffusion limitations compared to the extrudates. Additionally, the beads possessed a large amount of meso- and macropores, which is anticipated to be a favourable feature for application in CO_2_ adsorption because this should lead to fast adsorption/desorption kinetics.

To conclude, we synthesized a set of hierarchically porous beads containing Faujasite zeolite domains, which are promising adsorbents for CO_2_ separation from biogas due to their accessible pore structure, good CO_2_ adsorption capacity, and good CO_2_/CH_4_ selectivity. Additionally, these beads are also attractive for the separation of CO_2_ from more dilute gas streams due to their relatively high enthalpy of adsorption. 

## Figures and Tables

**Table 1:** Yield and crystalline phase of the zeolitic beads and of the powder side-products.

Sample	Yield Beads (g)	Yield Powder (g)	Bead Diameter (mm)	Crystalline Phase(s) in the Beads	Degree of Crystallinity of the Beads (%)	Crystalline Phase in the Powder Side-Product
F1	0.22	2.73	0.59 ± 0.15	FAU	63	FAU
F2	0.21	2.72	0.59 ± 0.17	FAU + trace LTA	67	FAU
F3	0.19	2.97	0.50 ± 0.12	FAU + LTA	52	FAU

**Table 2:** Physicochemical properties of F1-beads–F3-beads, F1 powder side-product (F1-pow), commercial zeolite Y (NaY), and extrudates prepared from commercial zeolite Y (E-20%).

Sample	BET Surface Area (m^2^ g^−1^)	T-Plot Micropore Volume (cm^3^ g^−1^)	BJH Pore Volume (cm^3^ g^−1^)	Si/Al Ratio	Na/Al Ratio	Na-Content (mol g_material_^−1^)
F1-beads	614	0.19	0.41	2.4 ± 0.006	0.83 ± 0.01	0.183 ± 0.003
F2-beads	539	0.20	0.30	2.1 ± 0.084	0.71 ± 0.01	0.173 ± 0.001
F3-beads	550	0.18	0.37	2.5 ± 0.163	0.75 ± 0.09	0.162 ± 0.011
Commercial NaY	824	0.36	0.07	2.8 ± 0.048	0.92 ± 0.07	0.185 ± 0.014
E-20%	521	0.24	0.08	2.2 ± 0.056	-	-
F1-pow	639	0.26	0.54	1.5 ± 0.001	0.81 ± 0.03	0.244 ± 0.008

**Table 3:** CO_2_ and CH_4_ adsorption capacity and CO_2_/CH_4_ selectivity for all the studied adsorbents.

Sample	CO_2_ Adsorption (mmol g^−1^)		CH_4_ Adsorption (mmol g^−1^)		CO_2_/CH_4_ Selectivity ^a^
	at 1 bar CO_2_	at 0.4 bar CO_2_	at 1 bar CH_4_	at 0.6 bar CH_4_	
NaY	5.64	4.46	0.41	0.23	29.3
E-20%	4.09	3.16	0.24	0.16	28.8
F1-beads	4.04	3.42	0.45	0.30	17.0
F2-beads	4.31	3.65	0.46	0.30	18.3
F3-beads	3.82	3.22	0.38	0.25	19.1
F1-pow	5.15	4.41	0.57	0.36	18.2

^a^ CO_2_/CH_4_ selectivity was calculated using Sel. = (q_CO₂_/q_CH₄_)/(p_CO₂_/p_CH₄_), with q_x_ being the amount adsorbed at partial pressure p_x_ in the hypothetical gas mixture. A partial pressure of 0.4 bar was used for CO_2_ and 0.6 bar for CH_4_ to mimic biogas.

**Table 4:** Synthesis parameters for zeolites F1 to F3.

	Ageing	50 °C	100 °C
F1	7d	5d	4d
F2	8d	4d	2d
F3	8d	3d	2d

## Data Availability

Data are stored at the University of Groningen and can be shared upon request to the corresponding author.

## Supplementary Materials

The following supporting information can be downloaded at: https://www.mdpi.com/article/10.3390/molecules28052198/s1, Table S1: Volumes and areas calculated from the idealized framework model; Figure S1: XRD pattern of F1-beads; Figure S2: XRD pattern of F2-beads; Figure S3: XRD pattern of F3-beads; Figure S4: Deconvolution of XRD pattern of F2-beads; Figure S5: Deconvolution of XRD pattern of commercial NaY powder; Figure S6: SEM images of beads with less common features; Figure S7: XRD pattern of commercial zeolite NaY; Figure S8: Pore size distribution of the beads and powder samples; Figure S9: N2 physisorption isotherm of F1 powder side-product; Figure S10: Pore size distribution from the BJH desorption branch from 0–100 nm for F1 powder side-product; Figure S11: XRD pattern of F1 powder side-product; Figure S12: XRD pattern of F2 powder side-product; Figure S13: XRD pattern of F3 powder side-product; Figure S14: SEM image of F1 powder side-product; Figure S15: SEM image of F1 powder side-product. Zoomed in on one large grain; Figure S16: SEM images of individual crystals of: (A) commercial NaY powder, (B) F3-beads; Figure S17: Freundlich–Langmuir fit for CO_2_ adsorption isotherms of commercial NaY powder at 15 °C, 25 °C, and 35 °C; Figure S18: Freundlich–Langmuir fit for CO_2_ adsorption isotherms of F2-beads at 15 °C, 25 °C, and 35 °C; Figure S19: Isosteric enthalpy of adsorption of CO_2_ on commercial NaY powder; Figure S20: Isosteric enthalpy of adsorption of CO_2_ on F2-beads.

## Sample Availability

The samples are not available from the authors.
