# Supplementary material for: Binderless Faujasite Beads with Hierarchical Porosity for Selective CO2 Adsorption for Biogas Upgrading
Source: Molecules. 2023 Feb 27;28(5):2198. doi: 10.3390/molecules28052198 (PMC10004648; doi:10.3390/molecules28052198)
Supplement: Supplementary file 1 [file molecules-28-02198-s001.zip › molecules-2147067-supplementary.pdf]

Binderless Faujasite beads with hierarchical porosity for selective CO_2 adsorption for biogas upgrading

Dina G. Boer, Zahra Asgar Pour, Jort Langerak, Benny Bakker, Paolo P. Pescarmona*

Supplementary information

Table S1: Volumes and areas calculated from the idealized framework model. Data obtained from [1]*, calculated by* [2].

	FAU	LTA
**Framework density**	13.3 T/1000 Å^3	14.2 T/1000 Å^3
**Occupiable volume**	28.6%	23.1%
**Accessible volume**	27.4%	21.4%
**Occupiable area**	1398 m^2/g	1427 m^2/g
**Accessible area**	1211 m^2/g	1205 m^2/g
**Specific occupiable area**	1856 m^2/cm^3	2018 m^2/cm^3
**Specific accessible area**	1608 m^2/cm^3	1704 m^2/cm^3

The definition of the quantities in the Table can be found on the IZA website [1].

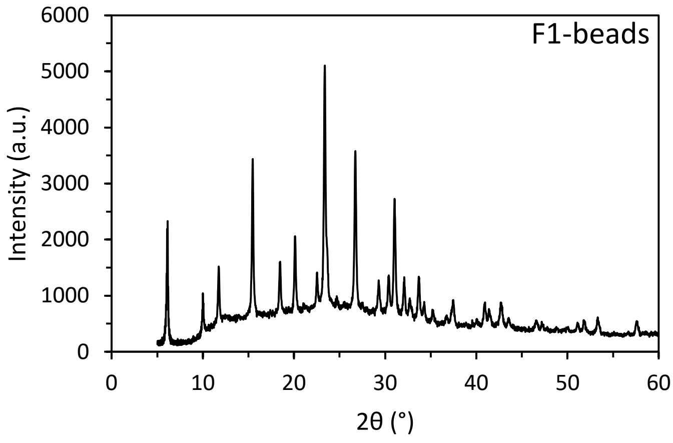

Figure S1: XRD pattern of F1-beads.

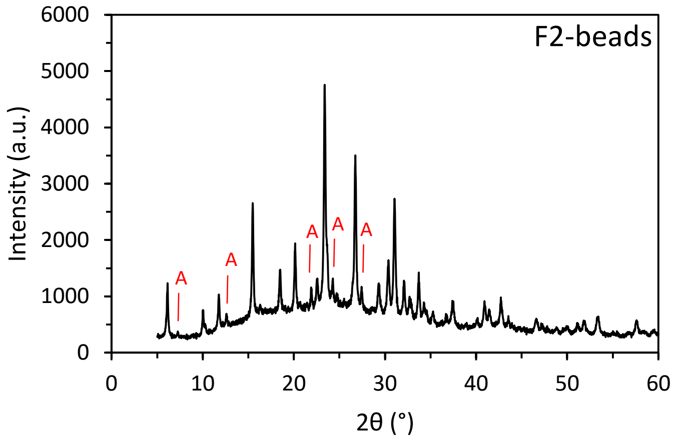

Figure S2: XRD pattern of F2-beads. Peaks indicated with an A correspond to framework type [LTA].

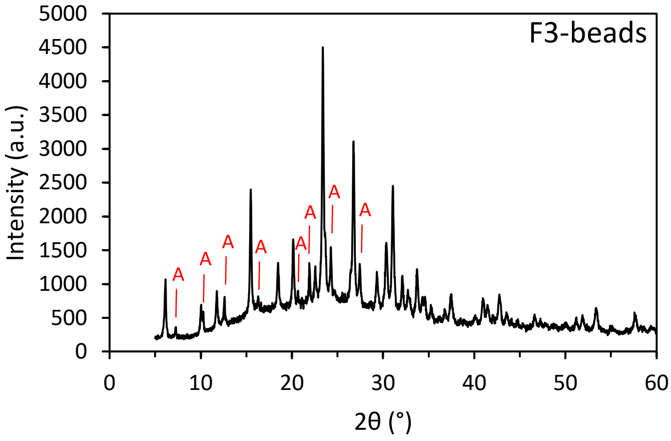

Figure S3: XRD pattern of F3-beads. Peaks indicated with an A correspond to framework type [LTA].

Estimation of the degree of crystallinity of the Faujasite beads

An estimation of the degree of crystallinity is obtained by deconvolution of the XRD diffractograms using the Fit Peaks function in OriginPro. Lorentzian functions were selected as peak type for the peaks corresponding to crystalline phases since this provides a better estimate of peak broadening. Gaussian functions were selected as peak type for the peaks corresponding to amorphous phases. The parameters were optimized to obtain a good fit ($R^2 \geq 0.98$). The degree of crystallinity was calculated by dividing the areas of all the peaks corresponding to crystalline phases by the total area of all peaks (see for example Figure S4). The calculated degree of crystallinity of the beads was normalized to that obtained from the highly crystalline commercial NaY powder (Figure S5) to account for background noise from the sample holder.

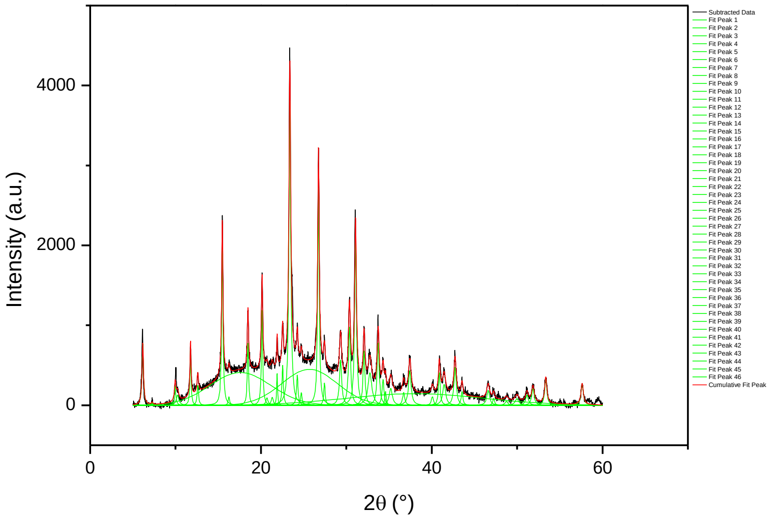

Figure S4: Deconvolution of XRD pattern of F2-beads.

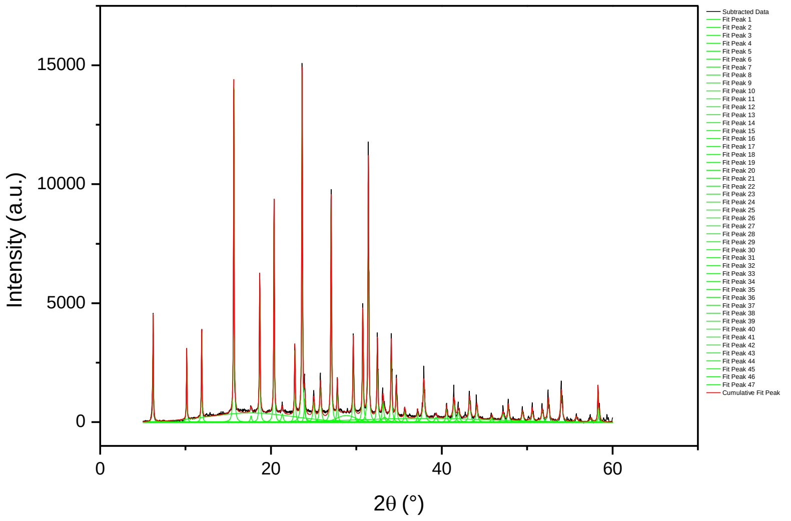

Figure 5: Deconvolution of XRD pattern of commercial NaY powder.

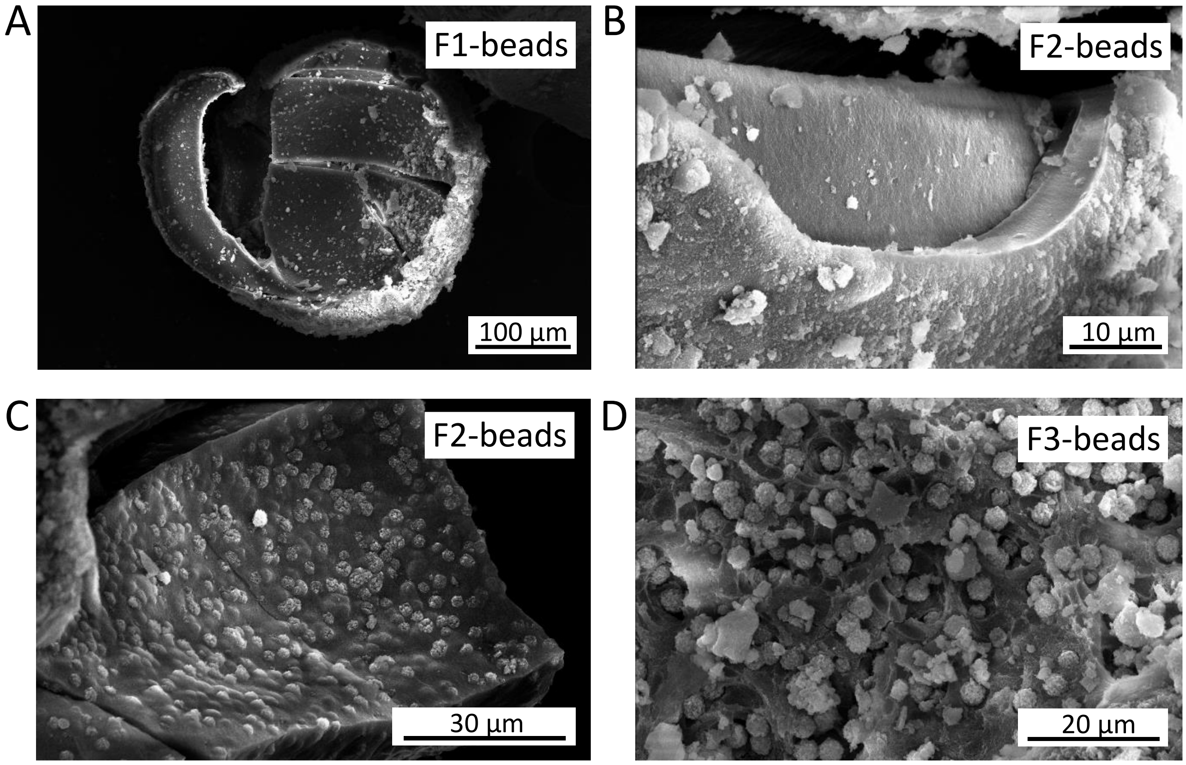

Figure S6: SEM images of beads with less common features: (A) an empty shell, (B) a bead with a clear distinction between the shell and the interior, (C) and (D) spherical aggregates of crystals that are embedded in an amorphous matrix.

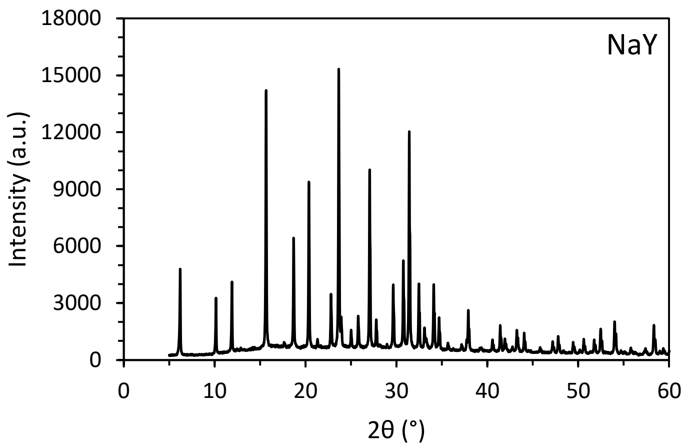

Figure S7: XRD pattern of commercial zeolite NaY.

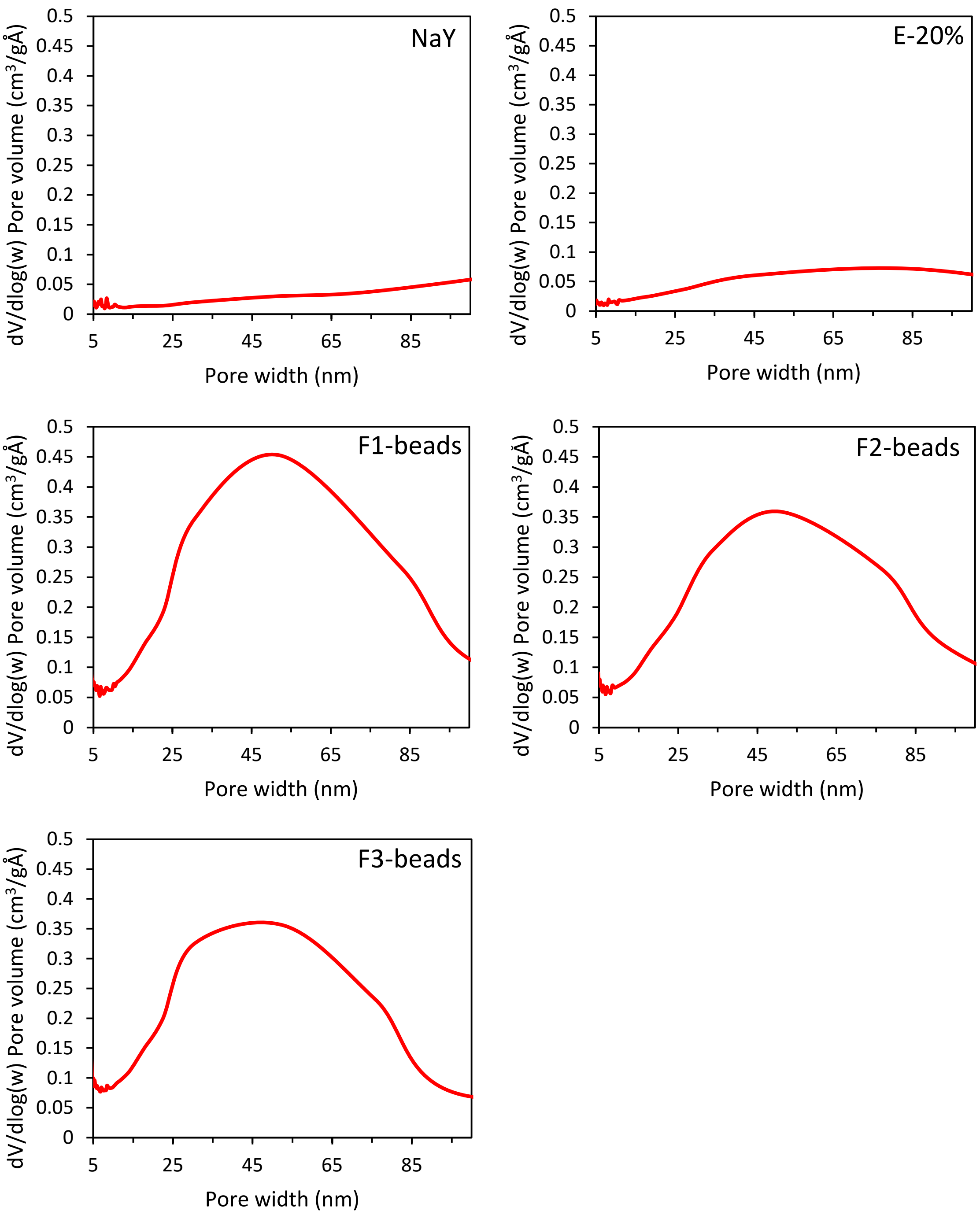

Figure S8: Pore size distribution of the beads and powder samples, determined by means of the BJH desorption branch from 5 to 100 nm.

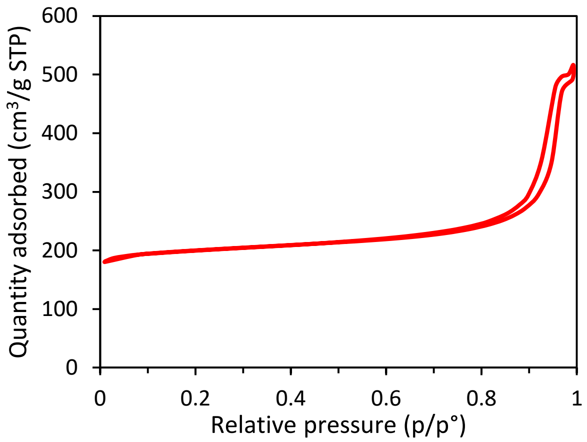

Figure S9: N_2 physisorption isotherm of F1-powder side product.

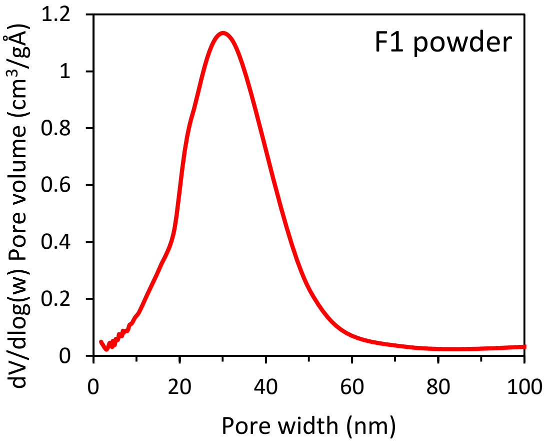

Figure S10: Pore size distribution from the BJH desorption branch from 0-100 nm for F1-powder side product.

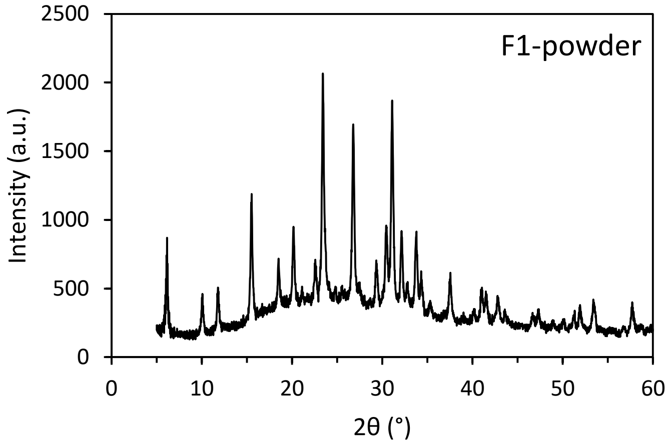

Figure S11: XRD pattern of F1 powder side product.

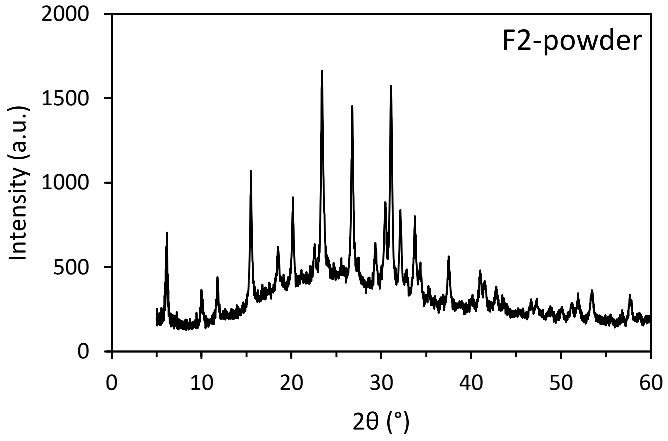

Figure S12: XRD pattern of F2 powder side product.

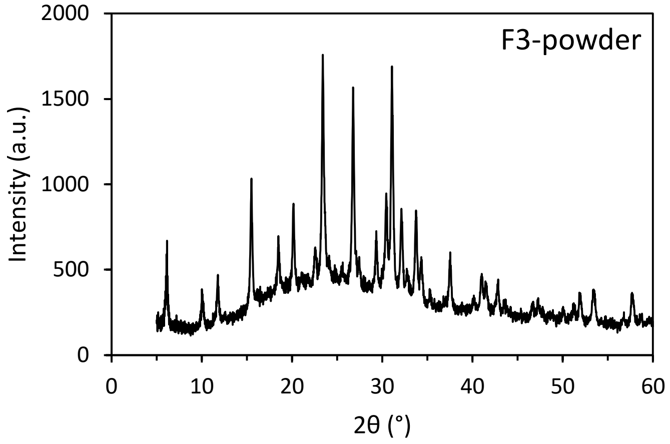

Figure S13: XRD pattern of F3 powder side product.

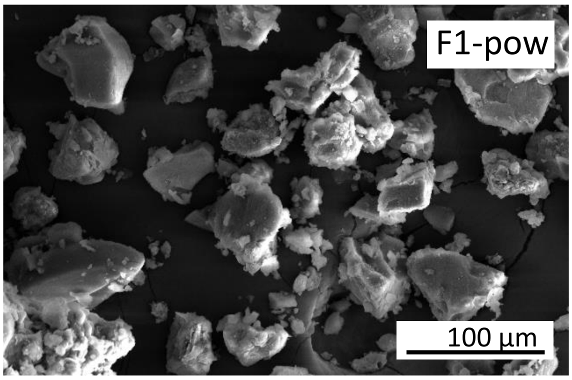

Figure S14: SEM image of F1 powder side product. The powder consists of large grains consisting of aggregated nano-sized crystals.

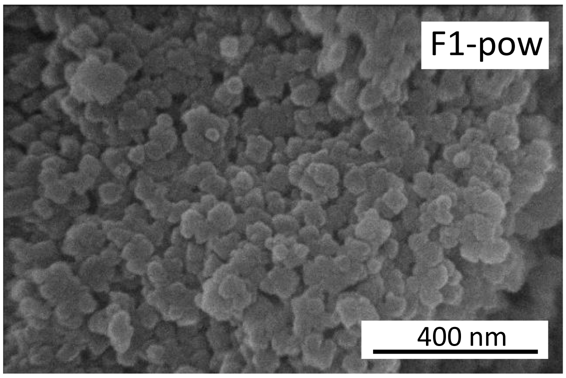

Figure S15: SEM image of F1 powder side product. Zoomed in on one large grain.

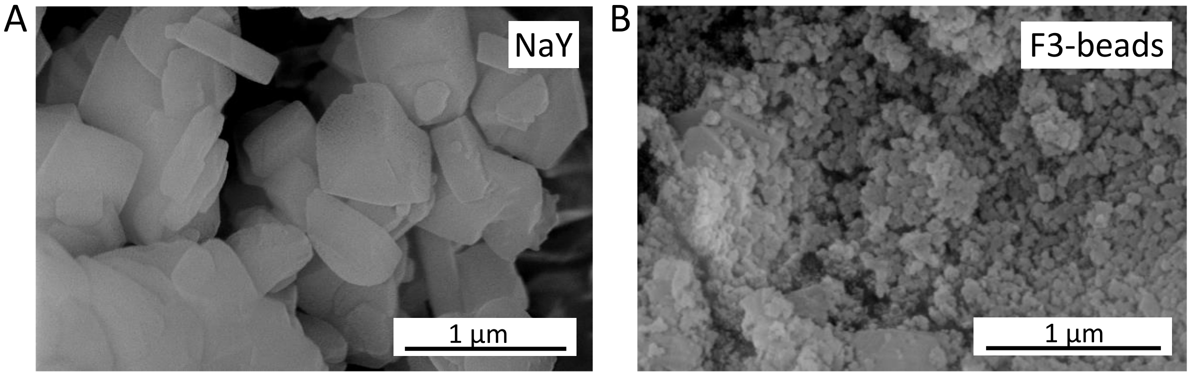

Figure S16: SEM images of individual crystals of: (A) commercial NaY powder, (B) F3-beads.

Calculation of the enthalpy of adsorption

The enthalpy of adsorption (ΔH_{ads}) was calculated using the Clausius-Clapeyron approach (detailed explanation of this method is given in ref. [3]).

First, the isotherms were fitted with the Freundlich-Langmuir eqn. (1):

$$n = \frac{a \cdot b \cdot p^c}{1 + b \cdot p^c} \tag{1}$$

In which n = adsorption capacity (mmol g^{-1}) p = pressure (kPa), a = maximal loading (mmol g^{-1}), b = affinity constant (1/kPa^c), c = heterogeneity exponent. Fitting the Freundlich-Langmuir equation in MATLAB using the experimental adsorption isotherms yielded the fitting coefficients a, b and c.

The Freundlich-Langmuir equation can be rearranged to:

$$p(n) = \sqrt[c]{\frac{n}{a \cdot b - n \cdot b}} \tag{2}$$

Using the Freundlich-Langmuir fit, pressure (p) – adsorption capacity (n) data pairs were generated with the same adsorption capacity n at each temperature. Subsequently, the data pairs are filled in the Clausius-Clapeyron equation to obtain the isosteric enthalpy of adsorption:

$$\Delta H_{ads}(n) = -R \cdot ln\left(\frac{p_2}{p_1}\right) \frac{T_1 \cdot T_2}{(T_2 - T_1)} \tag{3}$$

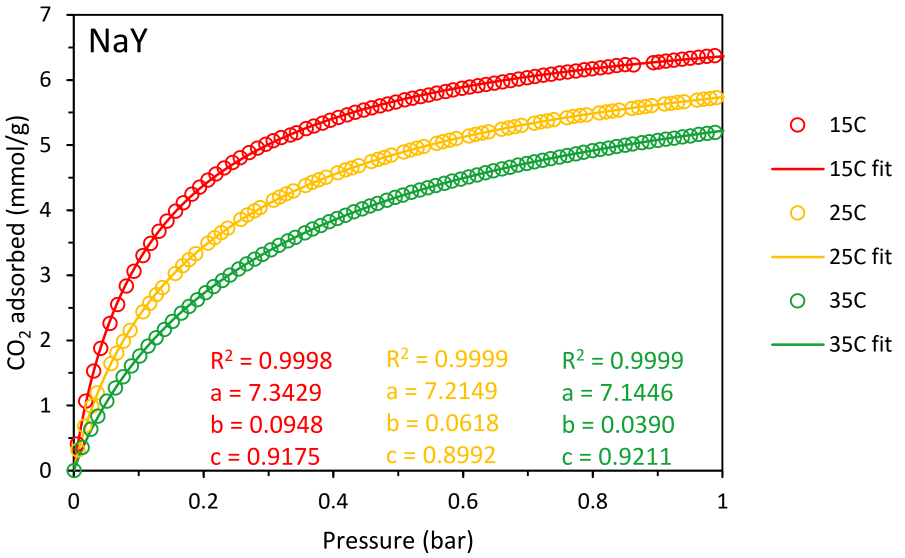

Figure S17: Freundlich-Langmuir fit for CO_2 adsorption isotherms of commercial NaY powder at 15°C, 25°C, and 35°C. Fitting parameters and R^2 values are shown in figure.

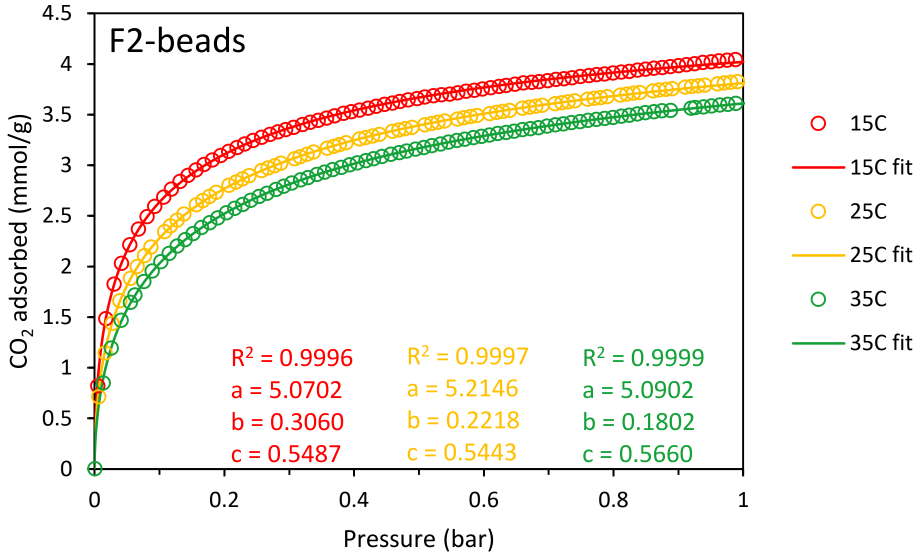

Figure S18: Freundlich-Langmuir fit for CO_2 adsorption isotherms of F2-beads at 15°C, 25°C, and 35°C. Fitting parameters and R^2 values are shown in figure.

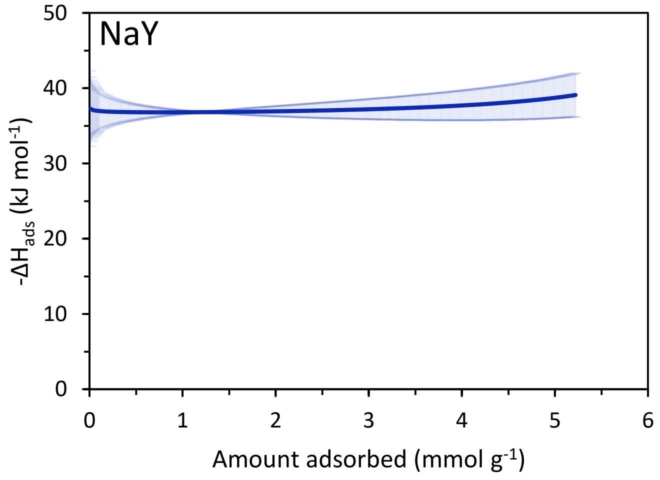

Figure S19: Isosteric enthalpy of adsorption of CO_2 on commercial NaY powder, derived from the CO_2 adsorption isotherms at 15°C, 25°C, and 35°C with standard deviation.

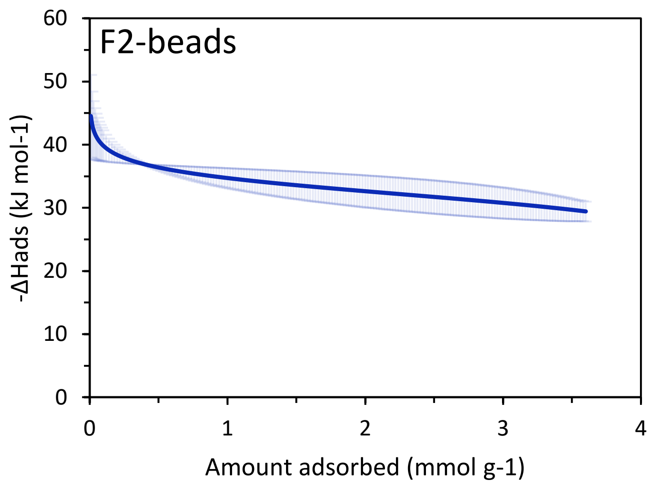

Figure S20: Isosteric enthalpy of adsorption of CO_2 on F2-beads, derived from the CO_2 adsorption isotherms at 15°C, 25°C, and 35°C with standard deviation.

References

[1] C. Baerlocher, L.B. McCusker, Database of Zeolite Structures, (2020). http://www.iza-structure.org/databases/ (accessed April 4, 2022). (This is reference 9 in the main text).

[2] M.D. Foster, I. Rivin, M.M.J. Treacy, O.D. Friedrichs, A geometric solution to the largest-free-sphere problem in zeolite frameworks, Microporous Mesoporous Mater. 90 (2006) 32–38. https://doi.org/10.1016/j.micromeso.2005.08.025. (This is reference 42 in the main text).

[3] A. Nuhnen, C. Janiak, A practical guide to calculate the isosteric heat/enthalpy of adsorption: Via adsorption isotherms in metal-organic frameworks, MOFs, Dalt. Trans. 49 (2020) 10295–10307. https://doi.org/10.1039/d0dt01784a. (This is reference 43 in the main text).
